# Comparative In Vitro Study of Sol–Gel-Derived Bioactive Glasses Incorporated into Dentin Adhesives: Effects on Remineralization and Mechanical Properties of Dentin

**DOI:** 10.3390/jfb16010029

**Published:** 2025-01-16

**Authors:** In-Seong Park, Hyun-Jung Kim, Jiyoung Kwon, Duck-Su Kim

**Affiliations:** 1 Department of Conservative Dentistry, School of Dentistry, Kyung Hee University, Seoul 02447, Republic of Korea; inseong0708@naver.com (I.-S.P.); kimhyunjung@khu.ac.kr (H.-J.K.); 2 Department of Conservative Dentistry, Kyung Hee University Dental Hospital, Seoul 02447, Republic of Korea; jykt55@naver.com

**Keywords:** dentin bonding, bioactive glass (BAG), sol–gel process, remineralization, elastic modulus, surface analysis

## Abstract

To overcome limitations of dentin bonding due to collagen degradation at a bonded interface, incorporating bioactive glass (BAG) into dentin adhesives has been proposed to enhance remineralization and improve bonding durability. This study evaluated sol–gel-derived BAGs (BAG79, BAG87, BAG91, and BAG79F) and conventional melt-quenched BAG (BAG45) incorporated into dentin adhesive to assess their remineralization and mechanical properties. The BAGs were characterized by using field-emission scanning electron microscopy (FE-SEM) and transmission electron microscopy for surface morphology. The surface area was measured by the Brunauer–Emmett–Teller method. X-ray diffraction (XRD) analysis was performed to determine the crystalline structure of the BAGs. Adhesive surface analysis was performed after approximating each experimental dentin adhesive and demineralized dentin by using FE-SEM. The elastic modulus of the treated dentin was measured after BAG-containing dentin adhesive application. The sol–gel-derived BAGs exhibited larger surface areas (by 400–600 times) than conventional BAG, with BAG87 displaying the largest surface area. XRD analysis indicated more pronounced and rapid formation of hydroxyapatite in the sol–gel BAGs. Dentin with BAG87-containing adhesive exhibited the highest elastic modulus. The incorporation of sol–gel-derived BAGs, especially BAG87, into dentin adhesives enhances the remineralization and mechanical properties of adhesive–dentin interfaces.

## 1. Introduction

Dentin is a humid porous biological composite in which the filler is composed of apatite crystals in a collagen matrix [1]. It is extremely difficult to bond owing to its humid and organic nature [2]. For dentin adhesion, a hybrid tissue at the resin–dentin interface is formed through the infiltration of monomer present in the adhesive into the collagen matrix. However, both etch-and-rinse and self-etching adhesives compromise bonding durability over time. Water plays an important role in the partial hydrolytic degradation of adhesive interfaces [3]. Collagen fibrils cannot be fully enveloped by monomers during the protocol; thus, perfect sealing might not be achieved at the resin–dentin interface. Furthermore, inactive proforms of proteolytic enzymes called matrix metalloproteinases (MMPs) have been identified in both mineralized and demineralized human dentin and exhibit collagenolytic and gelatinolytic activities due to the exposure of collagen fibrils when activated [4,5].

Several strategies have been introduced to overcome the degradation of the dentin bonding interface. Some of the approaches involve the use of inhibitors of proteolytic activity (e.g., chlorhexidine, galardin, tetracycline, and quaternary ammonium salts) [6,7,8,9], the increase in cross-linking agents (e.g., glutaraldehyde, ultraviolet-activated riboflavin, hesperidin, and proanthocyanidin) [10,11,12,13], and biomimetic remineralization [14,15]. The latter concept has been extensively studied. This study focuses on overcoming limitations and increasing the durability of dentin bonds. Resin-based adhesives containing fluoride, amorphous calcium phosphate, or bioactive glass (BAG) have been described to prevent the degradation of collagen in dental materials [16,17,18].

The first BAG, invented by Dr. Larry Hench, formed hydroxyapatite in aqueous solution and had it adhere to soft and hard tissues [19]. Hydroxyapatite formation proceeds as follows: BAG dissolves through hydrolysis and releases calcium and phosphate ions; then, the Si-O-Si bridge breaks, resulting in a SiO_2_-rich surface layer. Positively charged Ca^2+^ interact with negatively charged PO_4_^3−^ to form amorphous calcium in the surrounding fluid. The amorphous calcium phosphate forms hydroxyapatite [20]. Owing to this bioactivity, BAG is used in implants, dentin hypersensitivity, and air abrasion and is being tested for other applications [21]. The bioactivity of BAG varies with its size, shape, and composition. A previous study reported that nano-sized BAG accelerated hydroxyapatite formation more than conventional micro-sized BAG [22].

Sol–gel and melt quenching are two well-known methods for synthesizing BAG [23]. Compared with the conventional melt quenching method, the sol–gel method is advantageous because in the latter, the purity and composition of BAG can be controlled, a specific composition can be added, and the processing temperature is relatively low [24]. Recently, a highly ordered mesoporous BAG was synthesized to generate excellent bone-forming bioactivity in vitro [25]. Fiume et al. reported that BAG derived from sol–gel has a much larger surface area than the BAG obtained by melt quenching and exhibits better apatite-forming ability when the composition is identical [26].

Several studies have investigated the incorporation of BAGs into dental materials. Khvostenko et al. reported that resin composites containing BAGs reduced biofilm penetration into the marginal gap of restorations and prevented the progression of secondary caries [27]. Jang et al. reported that resin composites containing BAGs might exhibit a remineralization effect on adjacent demineralized dentin [28]. Tezvergil-Mutluay et al. reported that tooth remineralization can occur if BAG is added to an unfilled resin matrix [29]. Kim et al. reported that glass ionomer cements containing BAG exhibited fluorapatite formation on dentin surfaces without reducing the bond strength [30]. Although dental materials containing BAG as a bioactive component showed remineralization effects on adjacent tissue, it is necessary to focus on dentin adhesives to contain functional biomaterials for the remineralization of the closest interface to dentin. BAGs offer unique advantages over other approaches such as chemical MMP inhibitors, due to their ability to not only inhibit matrix metalloproteinases but also actively promote biomimetic remineralization, enhancing both the structural integrity and biological compatibility of dental tissues. Furthermore, BAGs are known for their cost-effectiveness and proven clinical safety, making them a preferable choice in restorative dentistry.

Thus, this study aimed to evaluate the effect of dentin adhesives containing sol–gel-derived BAG on remineralization and identify the optimal BAG composition and selection for dentin adhesives. The evaluation employed field-emission scanning electron microscopy (FE-SEM), transmission electron microscopy (TEM), X-ray diffraction (XRD), and elastic modulus measurements.

## 2. Materials and Methods

### 2.1. Materials

BAG45 (Sukgyung AT, Ansan, Republic of Korea) was synthesized by using the conventional melt quenching method and was used as a positive control. Four groups of sol–gel-derived BAGs, BAG79, BAG87, BAG91, and BAG79F, were prepared following the method described by Yun et al. [31]. The mesoporous BAGs were synthesized by using a modified sol–gel method under dilute conditions. In this process, cetyltrimethylammonium bromide was dissolved in a solution of distilled water and ammonium hydroxide, followed by the sequential addition of tetraethyl orthosilicate, triethyl phosphate, and calcium nitrate with vigorous stirring. The mixture was stirred for 3 h, filtered, and washed. The template was removed by calcination at 600 °C. To investigate the effect of silicon oxide (SiO_2_) content on nanosphere size, BAGs with varying SiO_2_ levels (BAG79, BAG87, BAG91, and BAG79F) were synthesized. A dentin adhesive (Any-Bond^TM^; MEDICLUS, Cheongju, Republic of Korea), which is not composed of any remineralizing components, was used as a negative control. Five experimental groups were prepared in which 3 wt% BAG was added to the dentin adhesive. Artificial saliva was prepared by dissolving calcium chloride (0.7 mmol/L), magnesium chloride hexahydrate (0.2 mmol/L), potassium dihydrogen phosphate (4.0 mmol/L), potassium chloride (30 mmol/L), sodium azide (0.3 mmol/L), and HEPES buffer (20 mmol/L) in distilled water [32]. The compositions of all the materials used in this study are listed in Table 1.

Sixty-nine caries-free human third molars were examined in this study. The included teeth were obtained from patients whose teeth were indicated for extraction, and informed consent was obtained from all patients. The experimental protocol using human teeth was reviewed and approved by the Kyung Hee University Dental Hospital Institutional Review Board (KH-DT21012), and all procedures were performed in accordance with the Declaration of Helsinki guidelines and regulations.

All the experimental groups are also listed in Table 2.

### 2.2. Methods

#### 2.2.1. BAG Characterization

Structural characterization was performed by FE-SEM (Apreo S; Thermo Fisher Scientific, Waltham, MA, USA) and TEM (JEM-1400 flash; JEOL, Tokyo, Japan). For FE-SEM, the BAGs were processed by using a platinum coating and examined at an operating voltage of 10 kV. For the TEM analysis, the BAGs were diluted in 100% ethanol and mixed thoroughly by using a vibrator. The BAGs were then dropped onto a 200–mesh grid. Drying was performed at an operating voltage of 120 kV.

The surface area of each BAG was measured by using the Brunauer–Emmett–Teller (BET) method with N_2_ gas adsorption.

#### 2.2.2. XRD Analysis

XRD analysis was performed to evaluate the apatite-forming ability of BAGs in an aqueous solution. Each BAG was stored in artificial saliva for 1 or 2 weeks. After storage, each BAG sample was rinsed with distilled water for 3 min and stored for 2 days in a digital incubator (TW-B110; Taewontech, Bucheon, Republic of Korea) for drying. XRD was performed three times (immediately and after 1 and 2 weeks of storage) by using an X-ray diffractometer (D8 Advance; Bruker, Billerica, MA, USA) under Cu Kα radiation of 40 kV with Ni filter. The diffraction intensities were measured by scanning in the range of 2θ (i.e., 20–50° in 0.01° steps for 0.1 s per step).

#### 2.2.3. Surface Analysis of Dentin Adhesives

To evaluate the surfaces of the dentin adhesives, six experimental groups were assigned (Table 2). Commercial composite resin (Any-ComTM; MEDICLUS, Cheongju, Republic of Korea) was placed in a silicon mold with dimensions of 6 × 6 × 4 mm^3^ to fabricate the specimen blocks. Three composite blocks were assigned to each group (N = 3). The top surfaces of the resin blocks were polished by using 600-grit silicon carbide paper. Each dentin adhesive was applied and light-cured with a LED curing light (SmartLite Focus; Dentsply Sirona, USA) according to the manufacturer’s instructions. They were stored in artificial saliva for 2 weeks and changed every 2 days. After storage, the samples were analyzed by using FE-SEM (Apreo S) coupled with energy-dispersive spectroscopy (EDS) (Xflash 6160; Bruker, Billerica, MA, USA).

#### 2.2.4. Surface Analysis of Bonding Interface Dentin

To investigate the changes in demineralized dentin, six experimental groups were assigned (Table 2). Completely demineralized dentin was used as the negative control (group DD). Three third molars were used from each group (n = 3). Dentin blocks with a volume of 5 × 5 × 2 mm^3^ were fabricated and completely demineralized by using a 4 N formic acid solution for 1 d. Subsequently, a composite resin block with dentin adhesive, as mentioned in Section 2.2.3, was prepared and approximated to the demineralized dentin surface as closely as possible by using an orthodontic rubber band. They were stored in artificial saliva for 2 weeks and changed every 2 days. After storage, the resin block was removed, and the dentin surface was washed with deionized water for 3 min. The dentin specimens were treated according to the process described by Perdigao et al. [33] and examined by using FE-SEM (Apreo S) and EDS (Xflash 6160).

#### 2.2.5. Measurement of Elastic Modulus of Dentin

Six third molars were used from each group. Dentin beam specimens (2 × 2 × 6 mm^3^) were prepared for each group. The sample size was determined according to the previous studies by using G Power [34,35]. An effective sample size of 6 subjects in each group would have a power greater than 0.80 (β = 0.2) with an α-level of 0.05. The dentin beams were completely demineralized with 4 N formic acid solution for 1 day. They were then treated in the same manner as described in Section 2.2.4. The elastic modulus of each group was assessed by using a 3-point bending test by a universal testing machine (AGS-X; Shimadzu, Tokyo, Japan). Additionally, the elastic modulus of the dentin beams after indirect application in each experimental group was assessed by using a 3-point bending test. The elastic modulus (E) was calculated by using the following equation: E = mL^3^/4 bh^3^, where m is the steepest slope along the linear portion of the load–displacement curve (N/mm), L is the span length (6 mm), b is the width of the test specimen (2 mm), h is the thickness (2 mm), and E is expressed in GPa [29].

#### 2.2.6. Statistical Analysis

The results of the elastic modulus were analyzed by using one-way ANOVA to determine statistical significance. Tukey’s HSD test was used for the post hoc comparison test. The level of significance was set to α = 0.05. Statistical analysis was performed using SPSS 23.0.0.0 (IBM Corp., Armonk, NY, USA) and GraphPad Prism 10.4.0 (GraphPad Software Inc., San Diego, CA, USA)

## 3. Results

### 3.1. BAG Characterization

**Figure 1:**
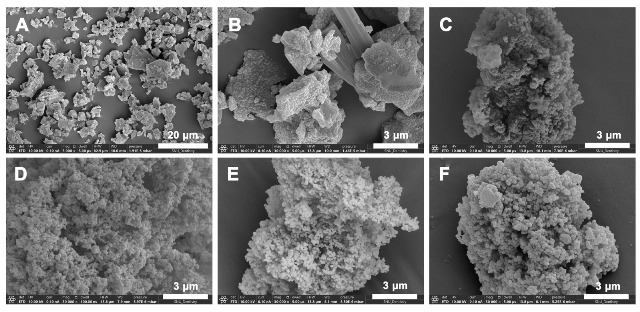
Representative FE-SEM images of BAGs. (**A**) BAG45 with low magnification (×5,000); (**B**) BAG45, (**C**) BAG79, (**D**) BAG87, (**E**) BAG91, and (**F**) BAG79F with high magnification (×30,000).

**Figure 2:**
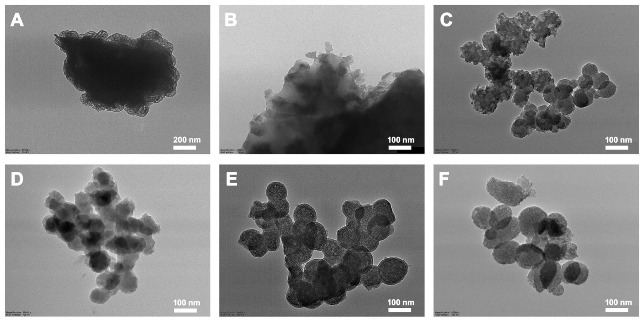
Representative TEM images of BAGs. (**A**) BAG45 with low magnification (×25,000); (**B**) BAG45, (**C**) BAG79, (**D**) BAG87, (**E**) BAG91, (**F**) and BAG79F with high magnification (×50,000).

Figure 1 and Figure 2 show the microstructures of the five BAGs obtained by using FE-SEM and TEM, respectively. The shapes and sizes of the BAGs differed. BAG45 exhibited clusters of large crystals of various sizes (Figure 1A,B and Figure 2A,B), and the four sol–gel-derived BAGs exhibited clusters of very small grains (Figure 1C–F and Figure 2C–F). The particle size of group BAG45 was large, making it difficult to observe with TEM at a high magnification (×50,000, Figure 2B), and the four sol–gel-derived BAG groups exhibited sizes of 50–100 nm (Figure 2C–F).

Table 3 presents the surface area measurements of the BAGs as determined by the BET method. The surface area of the four sol–gel-derived BAGs was approximately 400–600 times larger than that of BAG45. In particular, among the groups, BAG87 exhibited the largest surface area according to BET analysis.

### 3.2. XRD Analysis

**Figure 3:**
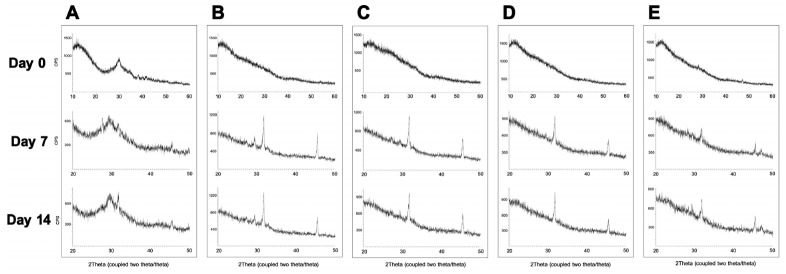
Representative XRD graphs of BAGs. (**A**) BAG45 (**B**) BAG79, (**C**) BAG87, (**D**) BAG91, and (**E**) BAG79F. After 7 days, BAG79, -87, and -91 exhibited 2–3 times more intense peaks at 29.5° compared with BAG45. After 14 days, BAG79, -87, and -91 also showed 2 times more intense peaks compared with BAG45 at 29.5° and 2–3 times at 45.5°.

Figure 3 shows the XRD patterns of the five BAGs at three different time intervals. For BAG45, the initial XRD pattern shows a prominent peak at 29.5°. At 7 and 14 days, additional peaks emerged at 31.8° and 45.5°, respectively. These peaks suggest the formation of crystalline phases such as calcite (CaCO_3_) at 29.5° and possibly hydroxyapatite or other calcium phosphate compounds at 31.8° and 45.5°, which are indicative of mineralization processes relevant to dental remineralization. In contrast, BAG79, -87, -91, and -79F (Figure 3B–E) synthesized via the sol–gel method initially exhibited no significant main peaks. However, peaks at 31.8° and 45.5° became apparent after one and two weeks, respectively, with intensities two–three times greater than those observed for BAG45. This significant increase in peak intensity suggests a more pronounced crystallization process, potentially indicating a higher rate of hydroxyapatite formation.

### 3.3. Surface Analysis of Dentin Adhesive

**Figure 4:**
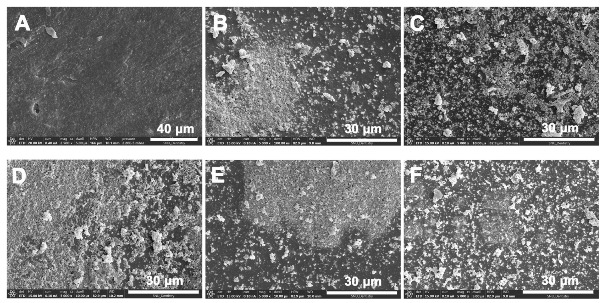
Representative FE-SEM images of adhesive surface. (**A**) Group DA; (**B**) DA45; (**C**) DA79; (**D**) DA87; (**E**) DA91; and (**F**) DA79F.

Figure 4 shows the change in the surface area of each dentin adhesive as observed by using FE-SEM. No precipitates were observed in the control group (Figure 4A). In contrast, amorphous precipitates were observed in all the experimental groups, but the mass of the precipitates was observed in group BAG45 (Figure 4B). However, more precipitates were observed in the four sol–gel-derived BAG-containing groups (Figure 4C–F). In particular, groups DA79, -87, and -79F exhibited extensive precipitates (Figure 4C,D,F). The results of the EDS analysis are presented in Table 4. The Ca/P ratio in each experimental group ranged from 1.40 to 1.76.

### 3.4. Surface Analysis of Demineralized Dentin

**Figure 5:**
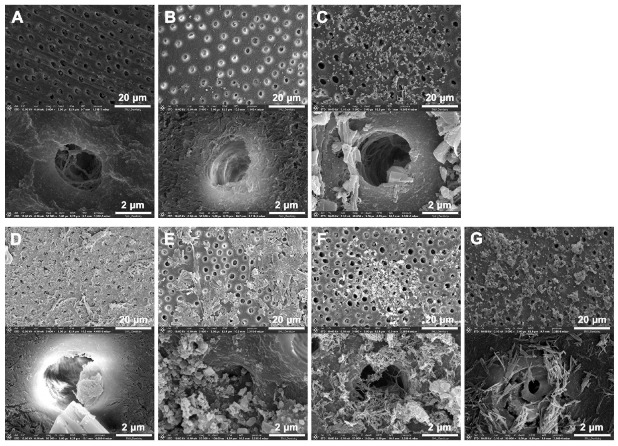
Representative FE-SEM images of dentin surface. (**A**) Group DD, completely demineralized dentin as control; (**B**) DA; (**C**) DA45; (**D**) DA79; (**E**) DA87; (**F**) DA91; (**G**) DA79F. Low magnification (×5000) of the experimental groups shown in the upper row and high magnification (×50,000) of the groups shown in the lower row.

Figure 5 shows the changes in the surface of completely demineralized dentin in all experimental groups. Demineralized dentin and groups DA exhibited no surface precipitates (Figure 5A,B). In contrast, group DA45 and the four sol–gel-derived BAG adhesive groups (DA79, -87, -91, and -79F) exhibited different kinds of precipitates on their surfaces (Figure 5C–G). DA79 and -79F exhibited precipitates on the dentin surface (Figure 5D,G), and BAG87 exhibited large flake-like crystalline precipitates, which were not observed in the other groups (Figure 5E). In contrast, some precipitates were observed in DA45 and DA91 (Figure 5C,F). The Ca/P ratio of all the experimental groups ranged from 1.12 to 1.80 (Table 4). Notably, the Ca/P ratio of mature human dentin is 1.62 [36]. No P ions were detected in group DA.

### 3.5. Measurement of Elastic Modulus

**Figure 6:**
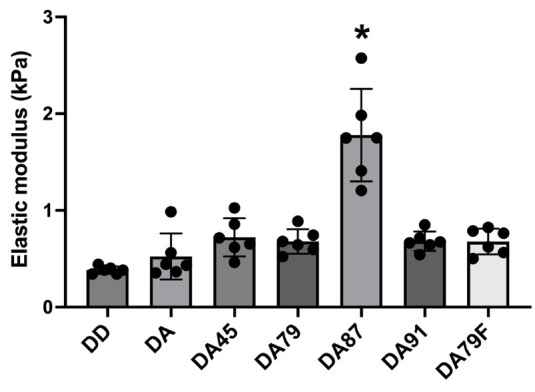
Elastic modulus measurement of dentin after experimental adhesive application. Asterisk (*) means statistical significance (*p* < 0.05).

Figure 6 shows the elastic moduli of all the experimental groups. After complete demineralization (group DD), the elastic modulus decreased significantly. The adhesive without BAG (group DA) did not increase the elastic modulus of demineralized dentin. After the approximation of the experimental BAG-containing adhesives, the elastic modulus increased; however, there was no statistically significant difference, except in group DA87 (*p* > 0.05). Group DA87 showed a significant increase in the elastic modulus of demineralized dentin (*p* < 0.05).

## 4. Discussions

BAG-containing dental materials are increasingly recognized as agents for tissue regeneration because of their ability to form apatite and facilitate the biomineralization of hard tissues. This study characterized different types of BAGs and evaluated their effectiveness when incorporated into dentin adhesives for remineralization.

Collagen degradation in the hybrid layer reduces the bonding durability at the adhesive interface and weakens the mechanical properties of dentin. BAG is a remineralization agent, and sol–gel-derived BAG can be incorporated into various dental materials owing to its nanoscale particle size. Some studies have been conducted on the remineralization effects of BAG-containing composite resins, or GIC [27,30,37,38,39]. However, in clinical situations, the composite resin does not directly come into contact with the tooth substrate, which limits its efficacy. In this study, BAG was incorporated into dentin adhesive to allow for direct interaction with demineralized dentin. We hypothesized that the void spaces within the dentinal tubules and the interface that did not form a hybrid layer could be filled by biomimetic remineralization. This approach aims to enhance the integrity of the adhesive interface by addressing the nanoleakage phenomenon [40]. To increase the remineralization efficacy of experimental dentin adhesives, this study investigated BAG characteristics, compared several types of BAGs, and suggested a rationale for proper BAG selection for incorporation into dentin adhesives.

Traditionally, BAGs have been synthesized by using high-temperature melt quenching processes, which limit control over their particle size and morphology [19]. This process does not permit the formation of nanoscale particles, and it poses challenges in controlling key properties of the resulting BAG. In addition, these BAGs usually exhibit irregular shapes and inhomogeneous particle size distributions [41]. In the 1990s, the sol–gel process emerged, providing a more versatile method for the design of BAG nanoparticles. Sol–gel technology allows for the synthesis of BAGs of comparable composition but at a much lower temperature. BAGs with a more controllable morphology and size can be achieved by using the sol–gel method, in which the precursors are mixed and allowed to react under liquid conditions in a controlled manner [42]. Thus, sol–gel-based methods are attracting increasing attention for the production of nanoscale BAGs, owing to their convenience and versatility. A significant advantage of BAGs produced via the sol–gel process is their excellent reactivity, which is attributed to the drastically increased surface area that can interact with the surrounding environment owing to their mesoporous characteristics.

In this study, the BAGs produced by sol–gel methods, such as BAG79, -87, -91, and -79F, exhibited surface areas 400–600 times greater than that of conventional BAG45, which might enhance their interaction with dental tissues and promote remineralization efficacy on larger interactive surfaces. While bone regeneration is primarily regulated by osteoblast signaling pathways, dentin regeneration is mainly achieved through mineral deposition. Unlike bone remodeling, dentin regeneration is a limited process. Consequently, it is more susceptible to physical environmental factors. In the context of dentin remineralization, a larger surface area facilitates increased ion exchange, leading to enhanced hydroxyapatite formation and more substantial mineral deposition.

The XRD analysis showed that the peaks changed after immersion in artificial saliva. This indicates that other substances were deposited on the glass surface. Initially, the broad peak observed at 29.5° for BAG45 indicates crystal deformation. The wider the peak, the smaller or less complete the crystal, indicating amorphous or more severe deformation in its properties [43]. Higher crystallinity appeared, and a sharp peak was observed after 1 and 2 weeks of immersion. The XRD patterns indicated the presence of hydroxyapatite crystals in all experimental groups, which is consistent with the results of a previous study [43]. These results were similar to those reported by Chen et al. [44]. The initial reaction of BAG45 was slow because the particles were impacted, but that of the sol–gel-derived BAG groups occurred quickly [22]. This indicates that the particles of the sol–gel-derived BAG groups have a mesoporous structure and are smaller than those of BAG45. Thus, the reaction area was wide, and hydroxyapatite and other calcium phosphate compounds formed more rapidly than BAG45. Balamurugan et al. reported the same reaction, in which minerals were formed around BAG after storage in aqueous solution [45].

Similar to the XRD analysis, the FE-SEM analysis revealed the significant presence of crystalline structures on both the adhesive surface and the demineralized dentin surface in the groups where adhesives containing BAG synthesized via the sol–gel method were applied. In all BAG-containing adhesive groups, numerous precipitates were observed in the FE-SEM images, with flake-like precipitates observed in group DA87. A previous study reported that flake-like precipitates have a higher Ca/P ratio than spherical apatite crystals [46]. The Ca/P ratio in demineralized dentin was more than 1.62 in group DA87. This indicates that after a significant drop in the Ca/P ratio due to dentin demineralization, contact with BAG increased the levels of mature human dentin.

The elastic modulus was measured to evaluate the mechanical properties of the dentin specimens. The elastic modulus of the experimental groups was higher than that of completely demineralized dentin. Although the increase in the elastic modulus of group DA87 was prominent, there was no significant difference among the experimental groups, except for group DA87. This was because a precipitate in DA87 with a higher Ca/P ratio was produced with a larger surface area, which led to a significant difference in the elastic modulus of dentin.

The crystallization exhibited in the sol–gel BAG groups is crucial for the mechanical enhancement of the dentin-adhesive interface. It is assumed that the deposition of crystalline structures in areas of nanoleakage, which inevitably occurs within the adhesive hybrid layer, strengthens the dentin–adhesive interface. Despite reports that incorporating more than 5% BAG into dentin adhesives can decrease adhesive strength and other mechanical properties, using sol–gel-derived BAGs, which have high surface areas, can mitigate these effects. The use of sol–gel-derived BAGs at nanoscales allows for enhanced remineralization at the dentin–adhesive interface while maintaining the same weight ratio, potentially without hampering the mechanical properties of the materials.

Although this study focused on the remineralization effect of BAG, it has been reported that the sol–gel process of BAG also exhibits regulatory effects on MMP activity, and several types of BAGs reduced MMP-2 and MMP-9 levels in a skin wound model [47]. The strengthening of the adhesive–dentin interface may not occur solely through the remineralization mechanism but could involve more complex interactions than our hypothesis suggests.

Our study demonstrated the superiority of BAG87 in an etch-and-rinse adhesive formulation by adjusting certain filler components, but these results may not be directly applicable to all dentin adhesive types. The pH level of adhesive systems can significantly influence the performance of BAGs, varying across self-etching, etch-and-rinse, and universal adhesives. Therefore, further studies are necessary to generalize these findings to other adhesive formulations.

This study was conducted by using indirect approaches, which involve observation by placing the adhesive surface in close proximity to demineralized dentin. Although this method allowed us to evaluate the remineralization potential, the direct observation of the hybrid layer structure in situ would provide a more comprehensive understanding of the adhesive interface. This limitation highlights the need for future studies to explore methods that enable the direct visualization of the hybrid layer to confirm and extend our findings. Another limitation of our study is the in vitro design, which, while allowing for the controlled observation of remineralization effects, does not allow for the full observation of the complex dynamics present in the oral cavity. Factors such as saliva, bacterial activity, and fluctuating pH play critical roles in the remineralization process and can affect the performance of adhesive materials. To address this limitation and better evaluate the clinical potential of BAG-containing adhesives, future research should include animal models or clinical trials. These study designs would provide more comprehensive insights into the adhesive performance and remineralization efficacy in vivo, thus facilitating the application of favorable in vitro results in the clinical practice of adhesive dentistry.

## 5. Conclusions

The incorporation of sol–gel-derived BAGs, especially BAG87, into dentin adhesives results in enhanced remineralization and mechanical properties of the adhesive–dentin interfaces.

## Figures and Tables

**Table 1:** Composition of materials.

Materials	Composition
BAG45	24.5%Ca, 6%P, 45%Si, and 24.5%Na
BAG79	16%Ca, 5%P, and 79%Si
BAG87	8%Ca, 5%P, and 87%Si
BAG91	4%Ca, 5%P, and 91%Si
BAG79F	13%Ca, 5%P, 79%Si, and 3%F
Artificial saliva	CaCl_2_ (0.7 mM), MgCl_2_·6H_2_O (0.2 mM), KH_2_PO_4_ (4.0 mM), KCl (30 mM), NaN_3_ (0.3 mM), and HEPES buffer (20 mM)
Dentin adhesive (DA)	bis-GMA, UDMA, HEMA, GPDM, photoinitiators, and EtOH

Abbreviations: BAG, bioactive glass; HEPES, hydroxyethyl piperazine ethane sulfonic acid; bis-GMA, bisphenol A-glycidyl methacrylate; UDMA, urethane dimethacrylate; HEMA, hydroxyethyl methacrylate; GPDM, glycerophosphoric acid dimethacrylate; EtOH, ethanol.

**Table 2:** Experimental groups.

Group	Description
DA	Dentin adhesive only
DA45	3wt% BAG45 added to dentin adhesive
DA79	3wt% BAG79 added to dentin adhesive
DA87	3wt% BAG87 added to dentin adhesive
DA91	3wt% BAG91 added to dentin adhesive
DA79F	3wt% BAG79F added to dentin adhesive

Abbreviation: BAG, bioactive glass.

**Table 3:** BET analysis of the five BAGs.

BAG Type	Surface Area (m^2^/g)
BAG45	1.0111
BAG79	572.2671
BAG87	665.0911
BAG91	441.8647
BAG79F	544.3679

Abbreviation: BAG, bioactive glass.

**Table 4:** Ca/P ratios of dentin adhesive and demineralized dentin.

Group	Ca/P Ratio	
	Dentin Adhesive	Demineralized Dentin
DA	–	– *
DA45	1.46	1.31
DA79	1.45	1.69
DA87	1.76	1.80
DA91	1.40	1.12
DA79F	1.64	1.22

* In group DA, the Ca/P ratio was unmeasurable due to the absence of phosphorus detection.

## Data Availability

The data presented in this study are available upon request from the corresponding author.
